# Predicting the outcome of grade II glioma treated with temozolomide using proton magnetic resonance spectroscopy

**DOI:** 10.1038/bjc.2011.174

**Published:** 2011-05-24

**Authors:** R Guillevin, C Menuel, S Taillibert, L Capelle, R Costalat, L Abud, C Habas, G De Marco, K Hoang-Xuan, J Chiras, J-N Vallée

**Affiliations:** 1 Department of Neuroradiology, Pitié-Sapêtrière Hospital, Functional Imaging Laboratory, INSERM U678, UPMC University Paris 6, 47-83 Boulevard de l’Hôpital, 75013 Paris, France; 2 Department of Neuro-oncology, Pitié Salpêtrière Hospital, 75013 Paris, France; 3 UPMC, UMI 209, UMMISCO, University of Paris 6, 75005 Paris, France; 4 IRD, UMI 209, UMMISCO, 93143 Bondy cedex, France; 5 Department of Neuroradiology, Pitié-Sapêtrière Hospital, 75013 Paris, France; 6 Department of Neuroradiology, XV-XX Hospital, 75571 Paris, France; 7 Laboratoire Contrôle Moteur et Mouvement, UFR STAPS Paris X, 200 Avenue de la République, 92001 Nanterre, France; 8 Department of Neuroradiology, Amiens University Medical Center, University of Picardie Jules Vernes, 80054 Amiens, France

**Keywords:** MRI, ^1^H-MRS, low-grade glioma, temozolomide, tumour response

## Abstract

**Background:**

This study was designed to evaluate proton magnetic resonance spectroscopy (^1^H-MRS) for monitoring the WHO grade II glioma (low-grade glioma (LGG)) treated with temozolomide (TMZ).

**Methods:**

This prospective study included adult patients with progressive LGG that was confirmed by magnetic resonance imaging (MRI). Temozolomide was administered at every 28 days. Response to TMZ was evaluated by monthly MRI examinations that included MRI with volumetric calculations and ^1^H-MRS for assessing Cho/Cr and Cho/NAA ratios. Univariate, multivariate and receiver-operating characteristic statistical analyses were performed on the results.

**Results:**

A total of 21 LGGs from 31 patients were included in the study, and followed for at least *n*=14 months during treatment. A total of 18 (86%) patients experienced a decrease in tumour volume with a greater decrease of metabolic ratios. Subsequently, five (28%) of these tumours resumed growth despite the continuation of TMZ administration with an earlier increase of metabolic ratios of 2 months. Three (14%) patients did not show any volume or metabolic change. The evolutions of the metabolic ratios, mean(*Cho*/*Cr*)_*n*_ and mean(*Cho*/*NAA*)_*n*_, were significantly correlated over time (Spearman *ρ*=+0.95) and followed a logarithmic regression (*P*>0.001). The evolutions over time of metabolic ratios, mean(*Cho*/*Cr*)_*n*_ and mean(*Cho*/*NAA*)_*n*_, were significantly correlated with the evolution of the mean relative decrease of tumour volume, mean(Δ*V*_*n*_/*V*_*o*_), according to a linear regression (*P*<0.001) in the ‘response/no relapse’ patient group, and with the evolution of the mean tumour volume (mean*V*_*n*_), according to an exponential regression (*P*<0.001) in the ‘response/relapse’ patient group. The mean relative decrease of metabolic ratio, mean(Δ(*Cho*/*Cr*)_*n*_/(*Cho*/*Cr*)_*o*_), at *n*=3 months was predictive of tumour response over the 14 months of follow-up. The mean relative change between metabolic ratios, mean((*Cho*/*NAA*)_*n*_−(*Cho*/*Cr*)_*n*_)/(*Cho*/*NAA*)_*n*_, at *n*=4 months was predictive of tumour relapse with a significant cutoff of 0.046, a sensitivity of 60% and a specificity of 100% (*P*=0.004).

**Conclusions:**

The ^1^H-MRS profile changes more widely and rapidly than tumour volume during the response and relapse phases, and represents an early predictive factor of outcome over 14 months of follow-up. Thus, ^1^H-MRS may be a promising, non-invasive tool for predicting and monitoring the clinical response to TMZ.

The WHO grade II gliomas (low-grade glioma (LGG)) accounts for 5–18% of all intracranial gliomas (Kleihues and Sobin, 2000). Although the optimal treatment for LGGs is currently the subject of debate, recent data suggest that temozolomide (TMZ; Brada *et al*, 2003; Hoang-Xuan *et al*, 2004) influences the evolution of LGGs (Mason *et al*, 1996; Chinot, 2001; Fortin *et al*, 2001). In patients who are suspected to have brain tumours, magnetic resonance imaging (MRI) is considered the gold standard for preoperative diagnosis. In addition, MRI is the optimal tool for gathering information for clinical decision making and monitoring (Dowling *et al*, 2001). Low-grade gliomas grow slowly and often appear as non-enhancing lesions, which makes it more difficult to assess their growth (Macdonald, 1994; Ricci and Dungan, 2001; Rollin *et al*, 2006). A volumetric evaluation may provide predictive arguments for patient outcome, as demonstrated by some authors (Swanson *et al*, 2003; Ricard *et al*, 2007). They performed a large study of the dynamic course of LGGs under TMZ treatment using mean tumour diameter (MTD) growth with a linear mixed model (Swanson *et al*, 2003; Ricard *et al*, 2007) that was extrapolated from serial T2-weighted images. Nevertheless, as the authors stated in their work, the MTD remains a morphologic parameter that requires from months to a year to provide reliable measure assessment. Some studies (Hoang-Xuan *et al*, 2004; Ricard *et al*, 2007) have suggested that chemotherapy may affect tumour burden without causing overt morphological modifications that can be visualised by MRI, possibly by affecting the sole infiltrative part of the glioma. The apparent response to TMZ, however, may appear to be delayed for several months if MRI-measured tumour volume is used as the principal parameter. Consequently, the biological behaviour of the glioma (e.g., whether it is stable or progressive) appears to be affected by the TMZ treatment (even at short delays).

Metabolic data obtained from proton magnetic resonance spectroscopy (^1^H-MRS) has proved valuable in therapy evaluation (Gill *et al*, 1990) and monitoring (Murphy *et al*, 2004). The aim of this prospective study was to evaluate the serial metabolic changes detected by ^1^H-MRS in LGGs treated with TMZ and to compare the results with the tumour volumes calculated by MRI. To our knowledge, this study is the first study to suggest that ^1^H-MRS can provide information for predicting the outcome for patients with LGG treated with TMZ at an earlier stage than conventional MRI volumetry.

## Materials and methods

### Patient selection

This study was a single-centre prospective study, and we used the following criteria.

*Inclusion criteria*

1. Patients with histologically confirmed LGG;
2. No oncological or steroid treatment before MRI examination;
3. No contraindication to MRI investigation;
4. Imminent initiation of first-line TMZ chemotherapy.

The decision was based on usual clinical (significant increase of seizures frequency, appearance of focal deficit) and/or radiological (volumetric, appearance contrast enhancement) criteria of tumour progression (Chinot, 2001; Hoang-Xuan *et al*, 2004; Ricard *et al*, 2007).

*Exclusion criteria*

1. Patients with radiological evidence of transformation into high-grade tumours, including marked contrast enhancement, oedema or necrotic areas.
2. Patients with histological evidence of transformation into high-grade tumours, including necrosis, mitotic figures, nuclear atypia and endothelial proliferation.

*Temozolomide treatment* All patients received TMZ at a dose of 200 mg m^−2^ per day for 5 consecutive days of a 28-day cycle. The dose and frequency were adjusted according to standard toxicity criteria. The intention was to give patients at least 12 cycles (up to 24) of TMZ, and treatment was continued until we observed disease progression or unacceptable toxicity. We only analysed the data collected during the first 14 months, because all patient data were available at this time.

### Magnetic resonance imaging

All patients underwent an MRI examination using a whole-body MRI (Signa 1.5T, General Electric Healthcare, Milwaukee, WI, USA) at 2 weeks before beginning the treatment and again after each chemotherapy cycle. The imaging evaluation included an anatomic MRI protocol, which used the following parameters: for T2-weighted coronal images, TR/TE 4500/100, matrix of 320 × 224 slice 3-mm slice thickness and 0-mm gap; for T2-FLAIR axial images, TI/TR/TE 2200/8800/140, matrix of 228 × 224, 3-mm slice thickness and 0-mm gap (bicallosal plane); for T1-weighted axial images, TR/TE 400/9, matrix of 512 × 224, 4-mm slice thickness and 0.4-mm gap. T1-weighted acquisition was repeated in the axial and coronal planes after intravenous administration of gadolinium. All sequences were acquired with identical positioning at each examination.

Tumour volumetry was evaluated by measuring the region of high-signal intensity on T2-FLAIR images with the same grey-level windowing using BrainVisa-Anatomist software (BrainVisa-Anatomist, CEA NeuroSpin, Saint Aubin, France; Cointepas *et al*, 2001), which allows for voxel-by-voxel segmentation processing (Menuel *et al*, 2005).

In this study, patients who demonstrated a continuous (from one examination to the next) decrease in tumour volume were considered responders in a ‘response’ phase. Patients who demonstrated (at any time during chemotherapy) a continuous increase in tumour volume were considered to be in a ‘relapse’ phase. Patients who showed no change in tumour volume were considered to be non-responders in a ‘no response’ phase.

The ^1^H-MRS data were obtained by point-resolved single-voxel spectroscopy (TR=1500 ms/TE=35/144 ms, 96 scans). In the initial examination, we used the same methodology as previously published (Guillevin *et al*, 2008). To sample the entire tumour, we used several voxels with a standardised volume of 6 cm^3^. Finally, only the voxel within the maximal value of Cho/Cr and Cho/NAA was registered and retained for the follow-up examinations. Voxel placement was performed by the same radiologist at each examination and was always in the same position for each patient. This position was confirmed to be within the tumour margins that were visible on T2-FLAIR-weighted images. The raw spectroscopic data were processed using Spectral Analysis General Electric software(General Electric Healthcare) for accurate quantification, and the data were normalised against the Cr resonance signal from the healthy contralateral symmetrical region. *N*-acetyl aspartate (2.02 p.p.m.), creatine (3.03 p.p.m.), choline (3.22 p.p.m.) and lactate (1.36 p.p.m.) resonances were assessed at the intermediate echo time (144 ms), and free lipid (0.8–1.2 p.p.m.) resonances were assessed at the short echo time (35 ms). We compared the changes over time in the Cho/Cr and Cho/NAA ratios and the resonances of free lipids and lactates relative to changes in tumour volume.

### Statistical analysis

*Variables measured over the evaluation period* Measurements of variables were taken before treatment (*n*=0 months) and repeated every month during the follow-up after treatment (*n*=1–14 months).

*Values for metabolic ratios* (*Cho*/*Cr*)_*n*_ and (*Cho*/*NAA*)_*n*_ at *n* months of follow-up (*n*=0 before treatment).

*Values for tumour volumes* *V*_*n*_ (tumour size)_*n*_ at *n* months of follow-up (*n*=0 before treatment).

*Response to treatment at n months of follow-up* (MRS response patterns)_*n*_: response/no relapse *vs* response/relapse *vs* no response.

(Response)_*n*_: (response *vs* no response)

(Relapse)_*n*_: (relapse *vs* no relapse)

*Variables tested over time* *metabolic ratios*: (*Cho*/*Cr*)_*n*_ and (*Cho*/*NAA*)_*n*_, *n*=number of months of follow-up (*n*=0 before treatment).

*Mean metabolic ratios*



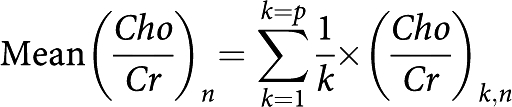





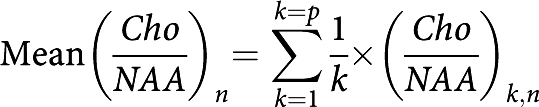



*n*=number of months of follow-up (*n*=0 before treatment); *p*=number of patients.

*Mean relative change in metabolic ratios* Mean relative change in metabolic ratios (*Cho*/*Cr*)_*n*_ and (*Cho*/*NAA*)_*n*_ at *n* months of follow-up compared with their corresponding reference ratio before treatment (*Cho*/*Cr*)_*n*_ and (*Cho*/*NAA*)_*n*_:



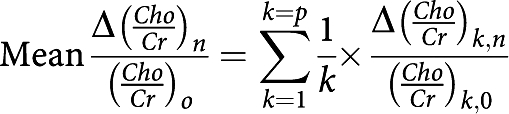



with



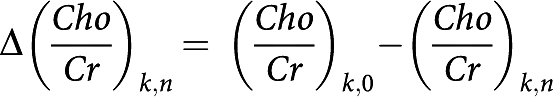





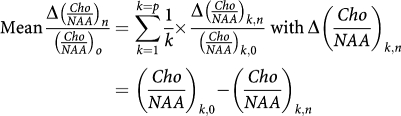



*n*=number of months of follow-up (*n*=0 before treatment); *p*=number of patients.

*Mean tumour volume variable*



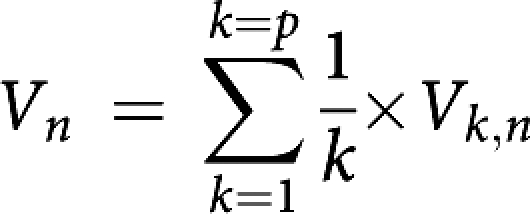



*n*=number of months of follow-up (*n*=0 before treatment); *p*=number of patients.

*Mean relative change in the tumour volume variable* Mean relative change in tumour volume, *V*_*n*_, at *n* months of follow-up compared with baseline tumour volume before treatment, *V*_0_:



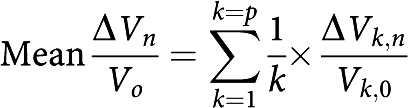



with Δ*V*_*k,n*_=(*V*_*k,*0_−*V*_*k,n*_); *n*=number of months of follow-up (*n*=0 before treatment); *p*=number of patients.

*Mean relative difference between two different metabolic ratios* Mean relative evolution of the difference between two different metabolic ratios, (*Cho*/*Cr*)_*n*_ and (*Cho*/*NAA*)_*n*_, compared with the reference ratio (*Cho*/*NAA*)_*n*_ at *n* months of follow-up:



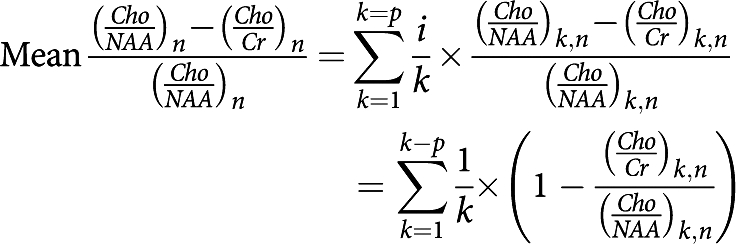



*n*=number of months of follow-up (*n*=0 before treatment); *p*=number of patients.

### Statistical methodology

- An initial univariate analysis was performed using Spearman's rank correlation coefficient to measure the statistical dependence between two metabolic ratio variables or between a metabolic ratio and a tumour volume variable. The analysis was then carried out using linear, polynomial, exponential and logarithmic regressions to test the temporal influence of the monotonic relationships observed between the metabolic ratios themselves and between the metabolic ratios and the tumour volume variables. The Wilcoxon test was used to assess comparisons of the differences between repeated measurements (quantitative variables) of the metabolic ratios and the tumour volume variables over time. To assess the significant differences between the spectral distributions of the metabolic ratio variables according to TMZ treatment response, the analysis was performed using the Kruskal–Wallis test for the MRS response patterns (qualitative variables with three classes), and the Mann–Whitney test was used to analyse the response and relapse variables (qualitative variables).
- A multivariate analysis was performed using the binary logistic regression test (qualitative variable) and multiple regressions (quantitative variable) using the LOGXACT programme (Cytel Software, Cambridge, MA, USA) to determine independent predictors of changes in tumour volume and predictors of response and relapse to treatment with TMZ.
- A receiver-operating characteristic (ROC) analysis was carried out to determine the cutoff for metabolic ratios that was predictive of response or relapse to treatment.
- Differences were considered statistically significant when *P*⩽0.05. Values are expressed as mean±s.d.

## Results

### Patient characteristics

The patient characteristics are presented in Table 1.

### Tumour volume and ^1^H-MRS analysis

All patients showed variations in their Cho/Cr and Cho/NAA ratios.

The results revealed three distinct ^1^H-MRS dynamic response patterns. The first pattern corresponded to responders/non relapsers: 13 patients (61.9%) responded to treatment without relapse. The second pattern corresponded to responders/relapsers: five patients (24%) briefly responded but then relapsed. The third pattern corresponded to non-responders: three (14%) patients did not demonstrated significant changes in tumour volume.

For two of the patients (patterns 1 and 2), whose maximal values of Cho/Cr and Cho/NAA were not observed in the same voxel, the voxel with the maximal value of Cho/Cr was used for the follow-up because this is the best tumour anabolism marker (Murphy *et al*, 2004; Lamari *et al*, 2008; Hlaihel *et al*, 2009).

Four patients showed an initial resonance of lactates: one patient was in pattern 1, one patient was in pattern 2 and two patients exhibited an additional resonance of free lipids (pattern 3).

**Figure 1:**
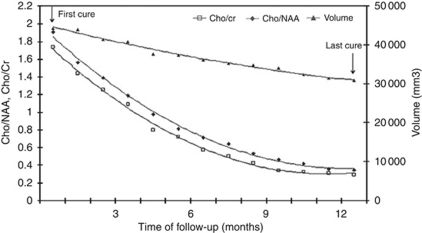
Evolutions over the time of the metabolic ratios and tumour volume in a patient treated with temozolomide, with a sustained response over 12 months without relapse. Note the great and rapid decrease of the metabolic ratios, compare with the low and delayed decrease of tumour volume.

Although the metabolite ratios and the volumetric parameter were consistent, the variability in the metabolite ratios was much greater (Figure 1);



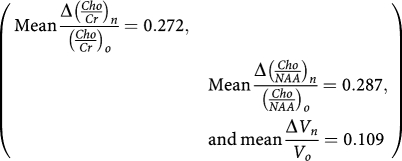



**Figure 2:**
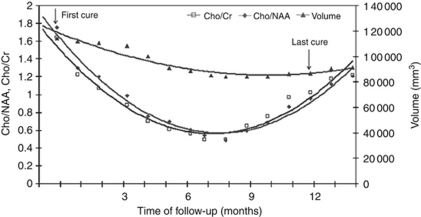
Evolutions over the time of the metabolic ratios and tumour volume in a patient treated with temozolomide, with a brief response and then relapse, over the time of follow-up. Note the great and rapid changes of the metabolic ratios in both phases, compare to the low and delayed change of tumour volume; (the minimum extremum points of the convex metabolic curves are observed at 8 month follow-up, and of the convex volumetric curve at 10 month follow-up).

**Figure 3:**
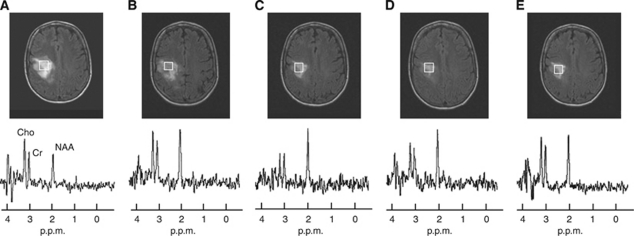
A series of FLAIR images from the patient from Figure 2, together with the spectrum at long TE. The data demonstrate a reduction in tumour volume during the response phase to temozolomide. The images displayed are (**A**) at 1 month, (**B**) 3 months, (**C**) 6 months, (**D**) 8 months after the first dose, corresponding to the tumoural metabolism activity before tumour regrowth, and (**E**) at 12 months.

In addition, for the five patients who relapsed early after their initial response, the minimum extremum points of the convex metabolite ratio curves occurred at 1 month (in three cases) and at 2 months (in the other two cases) before the minimum extremum point of the convex volumetric curve (Figures 2 and 3). The maximum mean relative decrease in tumour volume, mean(Δ*V*_*n*_/*V*_*o*_), was 0.183 at the 8-month follow-up, whereas the maximum mean relative decrease in the two metabolic ratios (mean(Δ(*Cho*/*NAA*)_*n*_/(*Cho*/*NAA*)_*o*_) and mean(Δ(*Cho*/*Cr*)_*n*_/(*Cho*/*Cr*)_*o*_)) were 0.560 and 0.515, respectively, and both were observed at the 6-month follow-up.

### Statistical analysis

**Figure 4:**
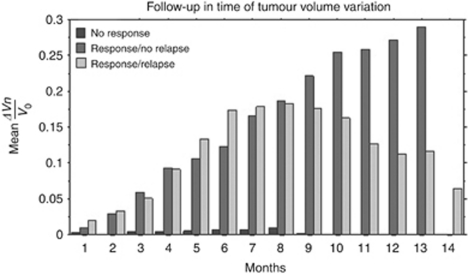
Mean relative change in tumour volume, mean(Δ*V*_*n*_/*V*_*o*_), for each pattern of response to temozolomide, over the period of follow-up.

*Tumour volume* The mean relative variation in tumour volume values (mean(Δ*V*_*n*_/*V*_*o*_)) over time is shown in Table 2, and the follow-up time was 14 months for each group (Figure 4).

**Figure 5:**
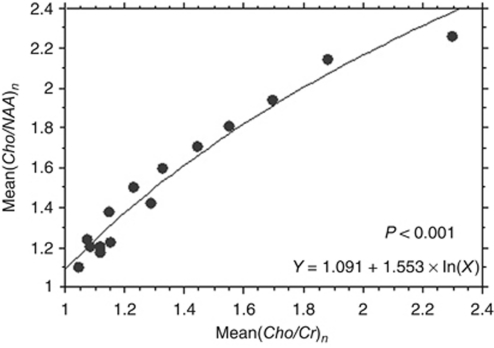
The evolutions of the metabolic ratios, mean(*Cho*/*Cr*)_*n*_ and mean(*Cho*/*NAA*)_*n*_, were significantly correlated over time (Spearman *ρ*=+0.95) and followed a logarithmic regression (*Y*=1091+1553 × ln(*X*); *R*^2^=0.926, *P*<0.001).

*The metabolic ratio curves* Spearman's rank correlation coefficient demonstrated, over time, a significant statistical monotonic relationship between the two metabolic ratios (i.e., mean(*Cho*/*Cr*)_*n*_ and mean(*Cho*/*NAA*)_*n*_, in the 21-patient group, *P*<0.001) as well as in the subgroups stratified according to the ‘MRS response pattern’ variable (Table 3). Mean(*Cho*/*NAA*)_*n*_ decreased significantly when mean(*Cho*/*Cr*)_*n*_ decreased (Spearman *ρ*=+0.95) over the 14 months of follow-up, and the decrease followed a significant logarithmic regression (*P*<0.001) that was described by the equation *Y*=1.091+1.553 × ln(*X*), *R*^2^=0.926 (Figure 5).

Both lactate and free lipid resonances, which were only relevant for four patients, did not show any statistically significant changes.

*Tumour volume and metabolic ratio curves* Spearman's rank correlation coefficient showed over time a significant statistical monotonic relationship between the tumour volume variable (mean(Δ*V*_*n*_/*V*_*o*_)) and the metabolic ratios (mean(*Cho*/*Cr*)_*n*_ and mean(*Cho*/*NAA*)_*n*_) in the 21-patient group (*P*=0.034 and 0.008, respectively) and in the subgroups that were stratified according to the ‘MRS response pattern’ variable (Table 3). Mean(*Cho*/*Cr*)_*n*_ and mean(*Cho*/*NAA*)_*n*_ decreased significantly when the mean relative decrease in tumour volume (mean(Δ*V*_*n*_/*V*_*o*_)) increased (Spearman *ρ*=−0.750 and −0.933, respectively) over the 14 months of follow-up.

**Figure 6:**
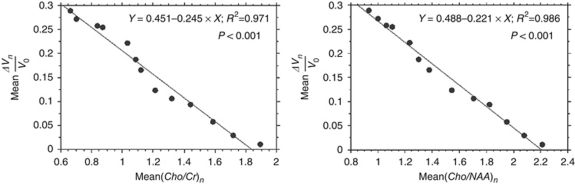
In the ‘response/no relapse’ patient group, the evolutions over time of metabolic ratios, mean(*Cho*/*Cr*)_*n*_ and mean(*Cho*/*NAA*)_*n*_, were significantly correlated with the evolution of the mean relative decrease of tumour volume, mean(Δ*Vn*/*V*_*o*_), according to a linear regression (*Y*=0451–0245 × *X*, *R*^2^=0971, *P*<0.001 and *Y*=0488–0221 × *X*, *R*^2^=0986, *P*<0.001, respectively).

In the ‘response/no relapse’ patient group, the evolutions of metabolic ratios, mean(*Cho*/*Cr*)_*n*_ and mean(*Cho*/*NAA*)_*n*_, were significantly correlated with the evolution of the mean relative decrease of tumour volume, mean(Δ*V*_*n*_/*V*_*o*_), over time (*P*<0.001), according to a linear regression that was described by the equations *Y*=0.451–0.245 × *X*, *R*^2^=0.971 and *Y*=0.488–0.221 × *X*, *R*^2^=0.986, respectively (Figure 6).

**Figure 7:**
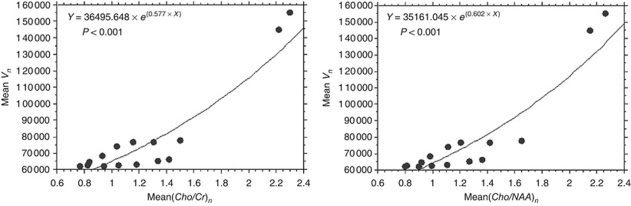
In the ‘response/relapse’ patient group, the evolutions over time of metabolic ratios, mean(*Cho*/*Cr*)_*n*_ and mean(*Cho*/*NAA*)_*n*_, were significantly correlated with the evolution of the mean tumour volume (mean*V*_*n*_), according to an exponential regression (*Y*=36495.648 × *e*^(0.577 × *X*)^, *P*<0.001 and *Y*=35161.045 × *e*^(0.602 × *X*)^, *P*<0.001, respectively).

In the ‘response/relapse’ patient group, the evolutions of metabolic ratios, mean(*Cho*/*Cr*)_*n*_ and mean(*Cho*/*NAA*)_*n*_, were significantly correlated with the evolution of the mean tumour volume (mean*V*_*n*_), over time (*P*<0.001), according to an exponential regression described by *Y*=36 495.648 × *e*^(0.577 × *X*)^ and *Y*=35 161.045 × *e*^(0.602 × *X*)^, respectively (Figure 7).

*The metabolic ratios curves and response to treatment* The mean metabolic ratios, mean(*Cho*/*Cr*)_*n*_ and mean(*Cho*/*NAA*)_*n*_, were significantly correlated with response to treatment over time (i.e., a 14-month follow-up) for the variable (MRS response patterns)_*n*_ (*P*=0.059 and 0.031, respectively, Table 2).

In addition, the mean relative change in the metabolic ratios, mean(Δ(*Cho*/*Cr*)_*n*_/(*Cho*/*Cr*)_*o*_) and mean(Δ(*Cho*/*NAA*)_*n*_/(*Cho*/*NAA*)_*o*_), were significantly correlated with response to treatment over time (i.e., a 14-month follow-up) for the variable (MRS response patterns)_*n*_ (*P*<0.001, Table 2).

The observed trend in the metabolic ratio curves (*Cho*/*Cr*)_*n*_ and (*Cho*/*NAA*)_*n*_ over time, particularly in the ‘response/relapse’ patient group, led us to develop a parameter to highlight the variable (relapse)_*n*_. Our analysis was based on the meaning of the evolution of tumour volume and metabolic ratios parameters over time while taking into account (1) the more rapid decrease in the metabolic ratio (*Cho*/*NAA*)_*n*_ and the faster increase in the metabolic ratio (*Cho*/*Cr*)_*n*_ at recurrence in patients receiving TMZ, and (2) the minimum extremum points of tumour volume and metabolic ratios curves and the intersection point between the metabolic curves over time. The parameter we developed was 
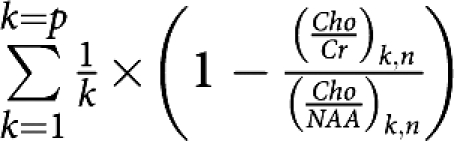
 where *n* is the number of months of follow-up (*n*=0 before treatment) and *p* is the number of patients.

It turns out that:



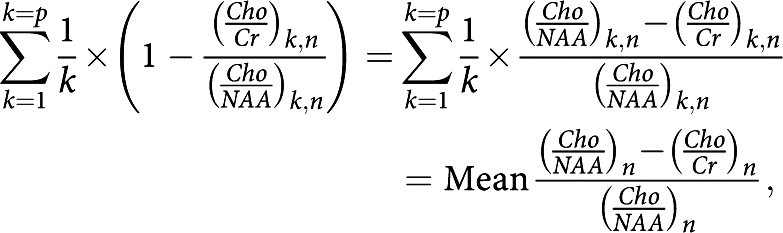



which was the mean relative difference between metabolic ratios (*Cho*/*NAA*)_*n*_ and (*Cho*/*Cr*)_*n*_ compared with the value of the reference ratio (*Cho*/*NAA*)_*n*_ at *n* months of follow-up. This parameter was well correlated with the variable (relapse)_*n*_ at *n* months of follow-up (*P*<0.001), and not with the variable (response)_*n*_ (*P*=0.541, Table 2).

*Multivariate study with respect to the variable (response)*_n_ The mean relative decrease of metabolic ratio, mean(Δ(*Cho*/*Cr*)_*n*_/(*Cho*/*Cr*)_*o*_), at *n*=3 months after starting treatment with TMZ, was an independent predictive factor of tumour response, (response)_*n*_, over the 14 months of follow-up (*P*=0.0364; mean(Δ(*Cho*/*Cr*)_*n*_/(*Cho*/*Cr*)_*o*_)=0.234±0.141). No significant cutoff for the mean(Δ(*Cho*/*Cr*)_*n*_/(*Cho*/*Cr*)_*o*_) was found for predicting the tumour response from the ROC analysis (Table 4).

*With respect to the variable (relapse)*_n_: The mean relative change between metabolic ratios, mean 
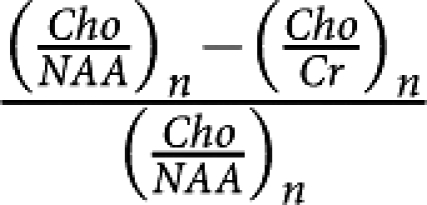
, at *n*=4 months after starting treatment with TMZ, was an independent predictive factor of tumour relapse, (relapse)_*n*_, over the 14 months of follow-up (*P*=0.058; mean 
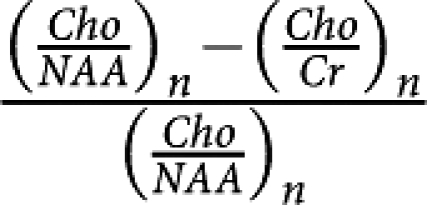
=0.134±0.110). A significant cutoff of 0.046 for mean 
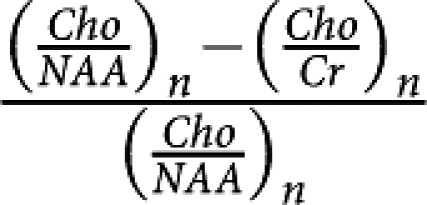
 with a sensitivity of 60% and a specificity of 100% (*P*=0.004) was found for predicting tumour relapse from the ROC analysis (Table 5).

This study was approved by the local ethics committee.

## Discussion

In general, our results are internally consistent. The metabolic ratio and volumetric curves were similar in evolution for both the response and relapse phases.

In each instance, our pretreatment spectroscopic data demonstrated higher choline and creatine levels and lower NAA levels relative to normal parenchyma. Moreover, five patients had additional resonances of lactates and/or free lipids, which agreed with the general metabolic profile of glial tumours reported by several groups (Negendank *et al*, 1996; Nelson *et al*, 1999). Immediately following the first month of treatment, however, we observed a clear, dramatic change in the metabolite ratios compared with tumour volume. The Cho/Cr and Cho/NAA ratios both decreased dramatically to the same extent, whereas tumour volume decreased much more slowly. This decrease in metabolic ratios was most likely a direct consequence of the therapeutic effect of TMZ and subsequent neoplastic cell death (Miller *et al*, 1996).

The second major finding of this study was a minimum extremum point in the metabolite curve, which appeared at 2 months in average before the same event registered on the tumour volume curve. This phenomenon was observed in all five patients with tumour recurrence, which suggests that ^1^H-MRS may be an earlier marker of tumour recurrence than volumetric data. In addition, this observation was consistent with the study of Tedeschi *et al* (1997). Moreover, a minimal change in tumour volume (registered using volumetric software in this study) may be difficult to assess with other methods, such as MTD, which could cause a delay in its assessment. Thus, ^1^H-MRS may provide additional time to optimise adjuvant therapy. After the intersection point between the metabolic curves, we observed a dramatic increase in both the Cho/NAA and Cho/Cr ratios. In addition, the range of variation of metabolite changes was much more wider than the variation in tumour volume in this part of the curve and in the response phase.

This difference between the metabolite ratios and the volume curves was not observed by either Murphy *et al* (2004) or Hlaihel *et al* (2009) who both reported that the metabolite ratios and tumour volume changed in parallel over time. In our opinion, this discrepancy emphasises the importance of being able to reproducibly position the voxel in the same precise location from one examination to another in the same patient, which we sought to achieve in this study. Indeed, our spectroscopic data were acquired using the same protocol at each examination for each patient. To the best of our knowledge, this study is the first multivariate analysis of spectroscopic data to provide predictive factors of LGG response during TMZ treatment. Indeed, the mean relative decrease in the Cho/Cr ratio slope at 3 months after initiation of TMZ chemotherapy was predictive of the response of the tumour over 14 months of follow-up. This last point emphasises the importance of using the Cho/Cr ratio slope as a tumour anabolism marker without regard to the degree of neuroaxonal tissue impairment. Cho/NAA was not a predictive factor for response, however, which was consistent with a previous study performed by (Hlaihel *et al*, 2009). In addition, the mean relative decrease between the Cho/Cr and Cho/NAA ratio slopes after 4 months of treatment was predictive of relapse over 14 months of follow-up, and their relative evolution immediately after treatment onset may be predictive of relapse over the follow-up period. Moreover, 0.046 was the significant cutoff of the predictive factor for the relapse variable, which has 100% specificity and 60% sensitivity. This value provides a reliable index of relapse risk that may be useful in clinical decision making. Conversely, no significant cutoff of Cho/Cr was found for the response variable. Although 14 months of follow-up may be considered too short by some authors (Murphy *et al*, 2004), it was a sufficient amount of time for early predictive factors of outcome to arise in this study.

The difference observed in this study between earlier and wider variations of the metabolite ratio parameters (Cho/Cr and Cho/NAA) and volumetric variations, both during the same short period, underlines the importance and complementary nature of these two measurements for monitoring the LGG response to TMZ. This difference holds true for both the response and relapse phases, and was consistent with the idea that metabolic changes both precede and determine morphological changes and tumour growth (Julia-Sape *et al*, 2006). The observation of both lactate and free lipid resonances may provide additional information regarding LGG metabolic behaviour, which we noted in a previous report (Lamari *et al*, 2008). The limited number of concerned patients (two) in this study, however, precluded our ability to draw any meaningful conclusions.

Methodologically, the heterogeneity of the metabolic behaviour of the tumour cannot be fully assessed by a single voxel measurement. As stated in the Materials and methods section, however, we sampled the entire lesion in our initial exploration, retained the most pejorative spectra, and then performed a study-to-study registration for voxel placement to minimise measurement errors that could lead to variability of biological factors.

Few authors have reported variability in the response to TMZ treatment (Chinot, 2001; Byrne, 2004). Some patients respond early (i.e., in the first month), whereas others react long after the first dose (i.e., after several months), which was the case in three of our patients in the ‘non-responder’ group. Likewise, timing of the relapse phase can also be variable. Hence, it is important to monitor patients over a long period (e.g., the entire TMZ treatment course) even though predictive factors may have been determined early in the follow-up, which was the case in this study.

## Conclusions

This study demonstrated concordance between ^1^H-MRS and tumour volume, which suggests that spectroscopy performed during MRI examination is valuable in the longitudinal follow-up of LGG that are being treated with TMZ. Our results indicate that the ^1^H-MRS profile of LGG (i) changes more widely and rapidly than tumour volume during both the response and relapse phases, (ii) is indicative of metabolite turnover before resumption in tumour growth and (iii) serves as an early predictive factor of outcome over a 14-month follow-up. Hence, ^1^H-MRS is a promising, noninvasive tool for predicting and monitoring the clinical response to TMZ.

## Figures and Tables

**Table 1:** Patient characteristics

	**Number of patients**	
**Characteristics**	***P*=21**	**%**
Age, median (range), years	43 (32–77)	
	
*Sex*		
Male	7	33
Female	14	67
		
*Histology*		
Oligodendroglioma	14	67
Oligoastrocytoma	5	24
Astrocytoma	2	9
		
Karnofsky score, median (range)	90 (80–100)	
		
*Surgery*		
Biopsy	12	56
Resection	9	44
		
Time interval from diagnosis to onset of TMZ, median (range), months	4 (1–84)	
		
*MRI (contrast enhancement)*		
Yes	3	14.7
No	17	81

Abbreviations: MRI=magnetic resonance imaging; TMZ=temozolomide.

**Table 2:**
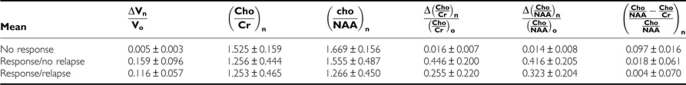
Mean (±s.d.) of metabolic ratios and response to treatment

**Table 3:**
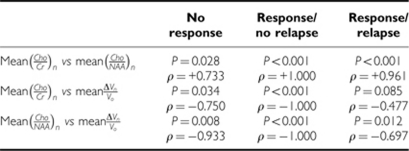
Spearman's rank correlation coefficients

**Table 4:** Receiver-operating characteristic analysis of *Cho/Cr* with respect to the variable ‘response’

**Cho/Cr**	**Cutoff**	**Sensibility (%)**	**Specificity (%)**	**Area**	***P***
Response	1.592	66.67	66.67	0.575	0.693

**Table 5:**
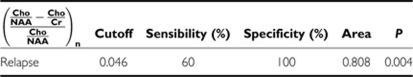
Receiver-operating characteristic analysis of (1−(*Cho/Cr/Cho/NAA*)_*n*_) with respect to the variable ‘relapse’
